# Multimeric ACE2-IgM fusions as broadly active antivirals that potently neutralize SARS-CoV-2 variants

**DOI:** 10.1038/s42003-022-04193-z

**Published:** 2022-11-12

**Authors:** Hristo L. Svilenov, Romina Bester, Julia Sacherl, Ramona Absmeier, Carsten Peters, Ulrike Protzer, Carsten Brockmeyer, Johannes Buchner

**Affiliations:** 1 Center for Protein Assemblies and Department Chemie, Technical University of Munich, Garching, Germany; 2 Faculty of Pharmaceutical Sciences, Ghent University, Ottergemsesteenweg 460, 9000 Ghent, Belgium; 3 Institute of Virology, Technical University of Munich/Helmholtz Zentrum Munich, Munich, Germany; 4 German Center for Infection Research, Munich partner site, Munich, Germany; 5 Formycon AG, Martinsried/Planegg, Planegg, Germany; 6 Brockmeyer Biopharma GmbH, Senator-Ernst Str. 2, Marzling, Germany

**Keywords:** Recombinant protein therapy, Antibody therapy, Recombinant protein therapy

## Abstract

Coronavirus infections are a world-wide threat to human health. A promising strategy to develop a broadly active antiviral is the use of fusion proteins consisting of an antibody IgG Fc region and a human ACE2 domain to which the viral spike proteins bind. Here we create antiviral fusion proteins based on IgM scaffolds. The hexameric ACE2-IgM-Fc fusions can be efficiently produced in mammalian cells and they neutralize the infectious virus with picomolar affinity thus surpassing monomeric ACE2-IgM-Fc by up to 96-fold in potency. In addition, the ACE2-IgM fusion shows increased neutralization efficiency for the highly infectious SARS-CoV-2 omicron variant in comparison to prototypic SARS-CoV-2. Taken together, these multimeric IgM fusions proteins are a powerful weapon to fight coronavirus infections.

## Abstract (web summary)

Fusion of ACE2 to IgM-Fc results in hexameric ACE2-IgM-Fc proteins with potent virus neutralization efficiency of patient-isolated SARS-CoV and SARS-CoV-2 variants.

## Introduction

Managing the ongoing pandemic caused by the severe acute respiratory syndrome coronavirus 2 (SARS-CoV-2) requires effective vaccines and antiviral drugs^1^. Monoclonal antibodies (mAbs) have proved to be efficient in neutralizing the virus and several products received emergency use authorization^2^. However, mAbs are very specific and vulnerable to escape mutations in new virus variants^3–5^. An orthogonal approach to antibodies employs soluble forms of the viral receptor, the angiotensin-converting enzyme 2 (ACE2), that can be used to “trap” and neutralize all coronaviruses that use ACE2 as a primary receptor^6–8^. To increase avidity, different fusion partners of ACE2 have been employed, including the Fc parts of IgG antibodies or oligomerization motifs^6,9–12^. As multi-valent binders seemed most promising, we exploited the potential of the oligomerization motif leading to the highest avidity in the immune system, the Fc segment of IgM^12^. It has been reported that reformatting virus-specific IgGs into IgM antibodies can lead to an enhancement in neutralization efficiency against SARS-CoV-2^13^. However, the increase in neutralization efficiency of these constructs varied greatly between different antibodies, and not every antigen-binding entity becomes significantly more potent when produced as an IgM^13^. Until now, it also remained unclear whether ACE2-IgM-Fc fusion proteins can be produced and whether such constructs will exhibit favorable properties such as hexamerization and enhanced virus neutralization properties.

## Results and discussion

**Fig. 1:**
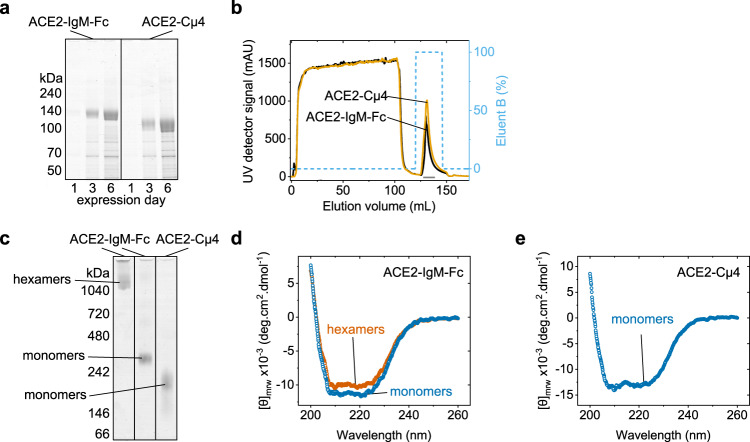
Expression and structure of ACE2-IgM fusions. **a** Reducing SDS-PAGE of cell supernatants after transient transfection of Expi293 cells. **b** Chromatograms from IgM affinity purification of cell supernatants. **c** Native PAGE of the purified proteins. **d**, **e** FUV CD spectra of the purified proteins.

To test the idea that the constant domains of IgM fused to ACE2 result in hexameric proteins with high neutralization capacity for the viral spike protein, we ligated the extracellular part of the human ACE2 (residues 18–740) via a flexible glycine-serine linker to the Fc domain (residues 106–453) of a human IgM^14^. Since it has been shown previously that the isolated C-terminal Cµ4 domain of IgM oligomerizes^15^, we also tested a construct where we fused ACE2 via the same linker to Cµ4 alone (residues 324–453). We produced the proteins by transient transfection in mammalian cells. Both ACE2-IgM-Fc and ACE2-Cµ4 were secreted well (Fig. 1a). After IgM affinity chromatography (Fig. 1b), the main fraction of ACE-Cµ4 eluted as one peak in size-exclusion chromatography (SEC), while ACE2-IgM-Fc eluted in two peaks corresponding to monomers and hexamers. The yield of purified ACE2-IgM hexamer was 20–25 mg/L which is comparable to marketed therapeutic IgG antibodies such as infliximab and pertuzumab when they are produced by transient transfection^16^. The peak fractions were further analyzed by native PAGE (Fig. 1c). The monomeric fractions of ACE2-Cµ4 and ACE2-IgM-Fc show bands at ~220 kDa and ~300 kDa, respectively. These molecular masses are close to the expected values for the respective monomers composed of two covalently connected polypeptide chains. The hexamer fraction of ACE2-IgM-Fc shows a band with a mass larger than 1000 kDa. Far-UV circular dichroism (FUV CD) spectra of the proteins revealed that they have similar secondary structures (Fig. 1d, e). All in all, these results show that ACE2 can be successfully fused to IgM constant domains and that their structural properties are retained.

**Fig. 2:**
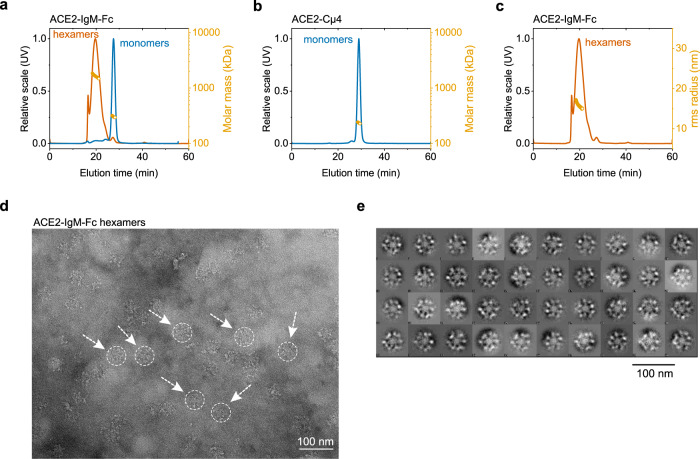
Oligomeric state of ACE2-IgM fusions. **a**, **b** SEC-MALS chromatograms with calculated molecular masses. **c** SEC-MALS chromatogram with calculated rms radius. **d** Representative TEM image of ACE2-IgM-Fc hexamers. **e** Class averages of the ACE2-IgM-Fc hexamers observed in TEM.

To investigate the oligomeric structure of the constructs further, we used SEC-MALS. The ACE2-IgM-Fc hexamer elutes as one main peak (≥90 %) exhibiting an average molecular mass of 1712 ± 6 kDa (Fig. 2a). The ACE2-IgM-Fc monomer peak gave a mass 297 ± 13 kDa (Fig. 2a). Thus, the molecular mass of the ACE2-IgM-Fc hexamer fits to a complex composed of six monomeric units. The ACE2-Cµ4 is monodisperse with M_m_ = 235 ± 1 kDa (Fig. 2b). Due to the large size of the ACE2-IgM-Fc hexamer, we were also able to calculate the average root mean square (rms) radius of the eluting protein which is 14.5 ± 0.1 nm (Fig. 2c). This radius is very close to the reported values for IgM hexamers^14^. We also observed that the relative fraction of the hexamer in the sample is not dependent on protein concentration (Supplementary Fig. 1).

To extend our analysis of the structural organization of the IgM-Fc hexamer, we performed negative-stain electron microscopy (Fig. 2d). The molecules observed resemble the typical hexameric IgM structure with a core ring formed by the Fc parts and the ACE2 domains pointing towards the outside of the oligomer (Fig. 2e). Thus, the fusion of ACE2 to the entire IgM Fc yielded multimeric molecules with an IgM-like architecture, while the fusion of ACE2 to Cµ4 did not result in oligomerization.

We were also interested in whether the thermal stability of the ACE2 domain is preserved in the ACE2 hexamer. Differential scanning calorimetry analysis revealed that the melting temperature of the ACE2 part is about 53.5 °C in both the ACE2-IgM-Fc hexamer and the ACE2-Cµ4 (Supplementary Fig. 2). This value is in line with the reported melting temperatures of other ACE2-Fc variants^17^. Therefore, the thermal stability of the ACE2 was not negatively affected by fusion to the IgM-Fc.

**Fig. 3:**
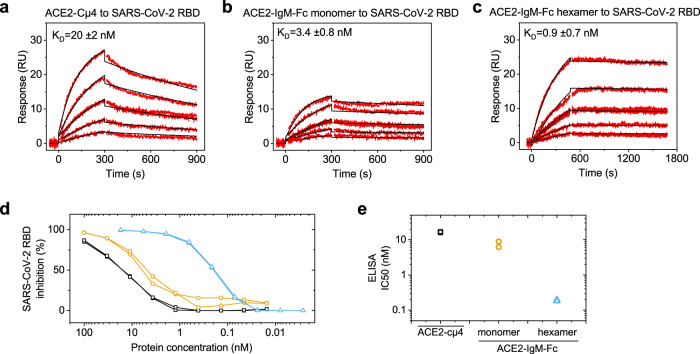
Antigen-binding properties of the ACE2-fusions. **a**–**c** Representative sensorgrams from SPR experiments for the binding of the ACE2-fusions to immobilized SARS-CoV-2 RBD on CM5 chips. Different concentrations of the ACE2-fusions were injected. The data were fit to a 1:1 binding model to obtain apparent dissociation constants. The dissociation constants are mean values of triplicates with standard deviations. **d** Binding of the ACE2-fusions to RBD determined by ELISA and the corresponding IC50s (*n* = 2). The lines are guide for the eyes. ACE2-Cµ4 (black squares), ACE2-IgM-Fc monomer (yellow circles), ACE2-IgM-Fc hexamer (blue triangles). **e** IC50 values (*n* = 2) from the fits to the ELISA data.

To test whether the hexameric ACE2-IgM-Fc binds to the isolated SARS-CoV-2 receptor-binding domain (RBD), we performed surface plasmon resonance (SPR) (Fig. 3a–c). It is important to note that the 1:1 binding model used to evaluate the SPR data provides only an apparent dissociation constant (K_D_) because the tested analytes are multivalent, and the binding is affected by avidity. For the ACE2-Cµ4 construct, we determined an apparent K_D_ of ~20 nM, while the ACE2-IgM-Fc monomer showed an apparent K_D_ of ~3.4 nM. Strikingly, the ACE2-IgM-Fc hexamer exhibited the most potent binding illustrated by an apparent dissociation constant of around 900 pM. An ELISA assay with immobilized full-length spike protein confirmed the SPR results (Fig. 3d). Here, the IC50s were 16 nM (ACE2-Cµ4), 7 nM (ACE2-IgM-Fc monomer) and 200 pM (ACE2-IgM-Fc hexamer), respectively in line with the trends observed by SPR (Fig. 3e).

**Fig. 4:**
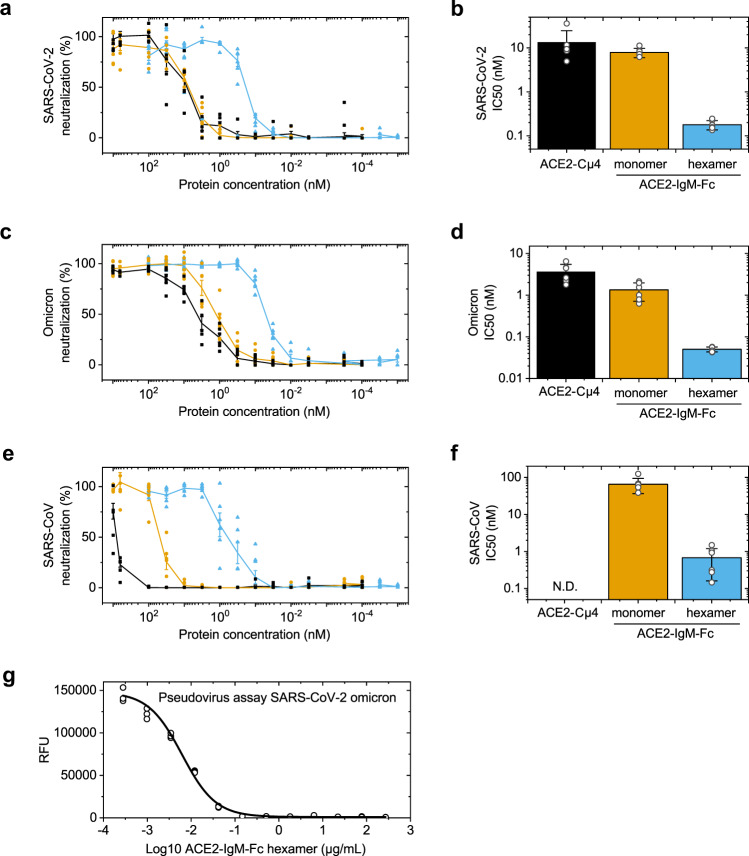
Virus neutralization properties by the ACE2-fusions. **a**, **c** and **e** Virus neutralization of SARS-CoV-2, omicron and SARS-CoV by the ACE2-fusions. The values are mean values with standard error of the mean (*n* = 6). Values from the individual replicates are shown. The lines are guide for the eyes. ACE2-Cµ4 (black squares), ACE2-IgM-Fc monomer (yellow circles), ACE2-IgM-Fc hexamer (blue triangles). **b**, **d** and **f** the corresponding IC50 values from the fit to the virus neutralization data. Mean values with standard deviation (*n* = 6). **g** Neutralization efficiency of ACE2-IgM-Fc hexamer in a commercial pseudovirus assay with the omicron variant. The IC50 from the fit is 6.6 ng/mL (95 % confidence interval 5.7 to 7.6 ng/mL). Individual values of triplicates.

While the binding assays using recombinant RBD and full-length spike proteins indicate the affinities for the two binding partners, they do not necessarily represent good proxies for inhibition of virus entry into human cells. To test this, we performed virus neutralization assays using infectious SARS-CoV-2 variants and Vero E6 cells. All constructs neutralized a SARS-CoV-2 variant isolated early in the pandemic (Fig. 4a and Table 1). The hexameric ACE2-IgM-Fc showed a remarkable enhancement in the infection neutralization efficiency (IC50 ~180 pM) compared to the ACE2-IgM-Fc monomer (IC50 ~7.9 nM) and ACE2-Cµ4 (IC50 ~13.5 nM) (Fig. 4b). This results in an approximately 44-fold lower IC50 for hexameric ACE2-IgM-Fc compared to the monomeric counterpart.

**Table 1:** Virus neutralization data for the constructs—Mean IC50s (nM) ±standard deviation (*n* = 6). The values are from the fits to the data in Fig. 4.

Construct	State	SARS-CoV-2	Omicron	SARS-CoV
ACE2-Cµ4	monomer	13.52 ± 11.03	3.66 ± 1.79	N.D.
ACE2-IgM-Fc	monomer	7.88 ± 1.88	1.34 ± 0.62	65.21 ± 28.74
ACE2-IgM-Fc	hexamer	0.18 ± 0.04	0.05 ± 0.01	0.68 ± 0.52
$\frac{({{{{{\rm{IC}}}}}}50{{{{{\rm{monomer}}}}}})}{({{{{{\rm{IC}}}}}}50{{{{{\rm{hexamer}}}}}})}$	-	44	27	96

These data raised the question of whether the ACE2-IgM-Fcs also show enhanced infection neutralization for SARS-CoV-2 variants of concern. We therefore tested the omicron variant that was shown to escape most of the available neutralizing antibodies (Fig. 4c and Table 1)^18^. Our ACE2-IgM-Fc hexamer neutralized the omicron variant with an IC50~50 pM (Fig. 4d). The monomeric ACE2-IgM-Fc and ACE2-Cµ4 constructs exhibited IC50s of ~1.3 nM and ~3.7 nM, respectively. Thus, the strong enhancement in virus neutralization by the hexameric ACE2-fusion also held true for the omicron variant.

Next, we were interested to determine whether other coronaviruses can be neutralized by the IgM-fusions. We therefore tested the infection neutralization efficiency of the constructs for the SARS-CoV that emerged in 2003 (Fig. 4e)^19^. Again, the hexameric ACE2-IgM-Fc exhibited very high neutralization efficiency illustrated by the IC50 value of 680 pM, while the monomeric ACE2-IgM-Fc had an IC50 of around 65 nM (Fig. 4f and Table 1), which was similar to a monomeric ACE2-IgG-Fc^8^. The ACE2-Cµ4 monomer was not able to fully neutralize SARS-CoV in concentrations up to 1000 nM (Fig. 4e).

Finally, to gain more insights into the virus neutralization potency of the ACE2-IgM-Fc hexamer, we performed a commercially-available pseudovirus neutralization assay employing ACE2-expressing HEK293 cells and pseudotyped lentivirus for the SARS-CoV-2 omicron with a luciferase reporter (Fig. 4g). Here we obtained an IC50 of 6.6 ng/mL (4 pM) which further testifies to the exceptional virus neutralization potency of the hexameric construct.

Taken together, the antigen-binding and infection neutralization data reveal an exceptional enhancement of antiviral activity (27- to 96-fold, Table 1) in the IC50s of the hexameric ACE2-IgM-Fc compared to their monomeric counterparts.

**Fig. 5:**
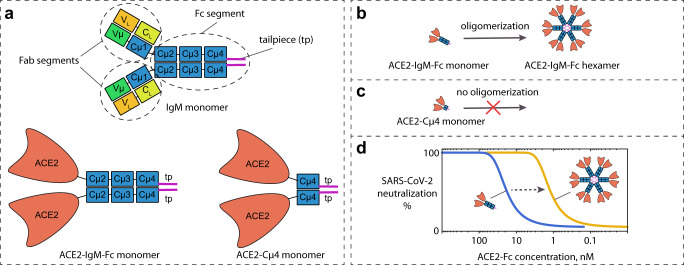
Structure of the ACE2-IgM-Fc and ACE2-Cµ4 fusion proteins and their oligomerization properties. **a** Structures of a human IgM monomer composed of two Fab segments and an Fc segment including a tailpiece, and monomeric ACE2-IgM-Fc and ACE2-Cµ4 composed of two identical polypeptide chains linked by disulfide bonds. **b** The ACE2-IgM-Fc monomer can oligomerize to form hexamers. **c** The ACE2-Cµ4 does not oligomerize. **d** The hexameric ACE2-IgM-Fc has more potent virus neutralization efficiency compared to its monomeric counterpart.

In summary, we have demonstrated that a hexameric ACE2-IgM-Fc protein can be efficiently produced and purified to homogeneity. The multimer fractions of recombinant IgM antibodies can contain both hexamers and pentamers^20^. Our analysis with native PAGE, SEC-MALS and TEM indicated a strong preference for hexamer formation by the ACE2-IgM-Fc. The hexameric ACE2-IgM-Fc exhibits much higher binding activity and infection neutralization efficiency of the original SARS-CoV, SARS-CoV-2 and its variants of concern compared to a monomeric ACE2-IgM-Fc and ACE2-Cµ4. A commercial pseudotype virus neutralization assay with the omicron variant confirmed the exceptional potency of the ACE2-IgM-Fc hexamer. Interestingly, as ACE2-Cµ4 did not form oligomers while Cµ4 alone forms hexamers^21^, it became obvious that fusion of the ACE2 domain to the entire IgM-Fc was required to obtain multimeric proteins with higher neutralization potency (Fig. 5). The enhancement in virus neutralization for the ACE2-IgM-Fc hexamer compared to the monomer observed in our study exceeded the enhancement observed by reformatting some neutralizing IgGs into IgMs indicating that the ACE2-IgM-Fc hexamer forms a favorable structure for antiviral fusion proteins^13^. Interestingly, reformatting IgG antibodies against SARS-CoV-2 as IgMs does not always result in proteins with significantly higher virus neutralization capacity^13^. Therefore, the large enhancement in the affinity of ACE2-fusions for the viral spike protein when expressed as multimeric IgM Fc-version is remarkable.

A wildtype sequence of ACE2 was used in this study due to the better thermal stability of this domain compared to an ACE2 with two mutations to abolish its enzymatic activity^17^. However, we have previously shown that ACE2 domains with no enzymatic activity can also be used to obtain ACE2-Fc fusions with minimal impact on the production yields, structure, and virus neutralization properties^8^. We therefore envisage that our approach can be employed to obtain hexameric ACE2-IgM-Fc proteins with enzymatically-inactive ACE2.

Until now, receptor fusion proteins comprising an Fc part from an antibody are mostly limited to the constant domains from IgGs^22^. Several strategies have been proposed to increase the binding avidity of ACE2-Fc (Table 2). These strategies rely on the fusion of multiple ACE2 sequences to an IgG scaffold^23^, employ a ligation to a p53 tetramerization domain^10^ or utilize *de novo* designed proteins that assemble Fc-fusions into nanocages^11^. In contrast, our approach takes advantage of the natural tendency of human IgM-Fc to form hexamers.

**Table 2:** Strategies to enhance the neutralization efficiency of ACE2-Fc fusion proteins based on increased binding avidity.

Engineering strategy	Nr of ACE2 domains	Assay	Fold increase in IC50	Source
Fc fusion to p53 tetramerization domain	4	pseudovirus	23	^10^
ACE2 fusion to C_L_ and C_H_1	4	pseudovirus	10–20	^23^
ACE2 fusion to C_L_, C_H_1 and the C-term of Fc	6	pseudovirus	11–30	^23^
Modular nanocages	24	pseudovirus	2.5–7	^11^
Fusion to IgM-Fc	12	patient-isolated virus	27–96	this work

A limitation of the current study is the lack of animal experiments. It will be important to define suitable animal models, administration routes, and clinical scenarios where the ACE2-IgM-Fc hexamer can bring the most benefits over other ACE2-fusions. Considering the importance of Fc-effector functions for the biological activity of ACE2-Fcs, it will be exciting to see how ACE2-IgM hexamers compare to ACE2-IgG-Fc constructs in terms of in vivo efficacy^24^.

Overall, the results obtained for the ACE2-IgM-Fc suggest that it may be possible and desirable to also combine other viral receptors with the IgM-Fc to obtain multimeric fusion proteins with excellent virus neutralization capacity. Such IgM-fusions could be particularly attractive for intranasal administration for the prevention or treatment of viral infections^13^.

## Methods

### Sequences

A signal peptide (MKWVTFISLLFLFSSAYS) at the N-terminus of each sequence was followed by the human ACE2 sequence (NCBI NP_001358344.1, residues 18-740), a linker (GGGSGGGS) and an antibody constant domain. The human IgM Fc (UniProtKB P01871, residues 106-453) and Cµ4 (UniProtKB - P01871, residues 324–453) sequences were used as fusion partners for the ACE2. The sequence of the SARS-CoV-2 receptor-binding domain (RBD) contained residues 319–541 (GenBank: QHD43416.1) with a 6xHis tag on the C-terminal. The genes encoding the sequences were obtained by commercial gene synthesis (GeneArt). A Kozak sequence and a stop codon were added between the HindIII and XhoI restriction sites. The nucleotide sequence was optimized for Homo sapiens with the GeneArt optimization tool. The gene inserts were delivered in the expression plasmid pcDNA3.1(+). Larger amounts of the plasmids were produced in XL-1 cells and purified with the PureYield™ plasmid midiprep system (Promega). The inserts in the plasmids were verified by commercial sequencing (Eurofins).

### Protein expression and purification

The proteins were transiently expressed with the Expi293™ expression system (Thermo Fisher Scientific). The purified plasmids were used for transfection following the manufacturer’s protocol for ExpiFectamine™ 293 transfection kit (Thermo Fisher Scientific). The amount of plasmid was 1 µg for 1 mL of cells. Six days after transfection, the cell supernatants were collected by centrifugation. All chromatographic purification steps were performed on an ÄKTA system (Cytiva). ACE2-IgM-Fc and ACE2-Cµ4 were first purified from the cell supernatants by affinity chromatography with the POROS™ CaptureSelect™ IgM affinity matrix (Thermo Fisher Scientific). The unbound sample was washed off with PBS. The bound proteins were eluted with cold 0.1 M glycine buffer pH 3.0 into 1/5 volume of cold 1 M Tris buffer pH 8.5. The His-tagged RBD was purified using a HisTrap HP column (Cytiva). The bound RBD was washed with 20 mM sodium phosphate pH 7.4 with 500 mM NaCl and 20 mM imidazole and eluted with 20 mM sodium phosphate pH 7.4 with 500 mM NaCl and 500 mM imidazole. After affinity chromatography, the ACE2 fusions were further purified and buffer-exchanged with size-exclusion chromatography on a HiPrep™ 16/60 Sephacryl® S-500 HR column (Cytiva) using PBS as the eluent. A HiLoad® Superdex 200 26/60 column (Cytiva) was used for to remove aggregates and exchange the buffer of the his-tagged RBD to PBS. Afterwards, the samples were flash frozen in liquid nitrogen and stored at −80 °C. Before the biophysical characterization and virus neutralization assays, the samples were thawed on ice, centrifuged and the concentration was measured by UV spectrometry using a calculated absorption coefficient (A_280nm,0,1%_) of 1.71 for ACE2-IgM-Fc, 1.87 for ACE2-Cµ4 and 1.31 for the SARS-CoV-2 RBD.

### Sodium dodecyl sulphate-polyacrylamide gel electrophoresis (SDS-PAGE)

The samples were mixed with 2× Laemmli buffer, loaded on 4–20% gradient gels (Serva) and ran with 50 mA current for 40 min. The gels were stained with colloidal Coomassie (Serva) for 2 h and destained in water overnight.

### Native PAGE

The samples were mixed with native PAGE loading buffer (ThermoFisher) and were analyzed on 3–8%, tris-acetate gels (NuPAGE™, ThermoFisher). The gels were run at 150 V for 2.5 h, stained with colloidal Coomassie (Serva) for 2 h and destained in water overnight.

### Size-exclusion chromatography coupled to multi-angle light scattering (SEC-MALS)

The system consisted of a Shimadzu HPLC, a HELEOS II MALS detector (Wyatt Technology) and a Superose® 6 Increase 10/300 GL column (Cytiva). PBS was used as a running buffer with a flow rate of 0.5 mL/min. Data acquisition was performed using the Astra software v5 (Wyatt Technology).

### Circular dichroism (CD)

A Jasco J-1500 spectropolarimeter was used for CD measurements. The spectra were obtained at 20 °C. Protein concentration was 0.1 mg/mL for far-UV measurements. The mean residue ellipticity for the buffer-subtracted CD spectra was calculated.

### Surface plasmon resonance (SPR)

A Biacore X-100 system (cytiva) was used to measure the binding affinities using HBS-EP + as the running buffer. The ligand (His-tagged RBD of SARS-CoV-2) was immobilized to around 100 RU on CM5 chips. The ACE2-fusion proteins were injected in multi-cycle kinetic mode. The injected concentrations for the monomeric ACE2-fusions were 12.5, 25, 50, 100, and 200 nM. The concentrations for the hexameric ACE2-IgM-Fc were 2.5, 5, 10, 20, and 40 nM. The sensorgrams were fit to a 1:1 binding model with the Biacore X-100 software. Although the 1:1 binding model may not ideally describe the binding of a multivalent analyte to the immobilized ligand, the provided apparent K_D_ values can be used for a first comparison between the constructs.

### ELISA using SARS-CoV-2 RBD

Inhibition of binding of SARS-CoV-2 RBD to ACE2 by the constructs was tested using the SARS-CoV-2 Surrogate Virus Neutralization Test Kit (GenScript; Cat.No. L00847-A) according to the manufacturer’s instructions. Briefly, samples were used pure or three-fold serial diluted with PBS and mixed with diluted HRP-RBD solution with a volume ratio of 1:1. After an incubation time of 30 min at 37 °C, 100 µl of the sample/RBD mixture was added to a 96-well plate precoated with the with human ACE2 and incubated for 15 min at 37 °C. The plate was washed with 260 µl 1× Wash solution and 100 µl 3,3′,5,5′-tetramethylbenzidine (TMB) solution was added to each well and incubated for 15 min at RT in the dark. The reaction was stopped by adding 50 µl of stop solution and absorbance was measured at OD_450_ on an Infinite F200 multi-plate reader (Tecan Group AG).

### Virus strains

SARS-CoV-2 (EPI_ISL_582134), SARS-CoV (NCBI:txid229992) and SARS-CoV-2 Omicron (EPI_ISL_7808190) were isolated from patient material in Germany. The viral strains were propagated and passaged on Vero E6 cells (ATCC-CRL-1586). DMEM medium supplemented with 10% fetal calf serum (FCS), 1% penicillin/streptomycin (P/S), 200 mmol/L L-glutamine, 1% MEM-Non-Essential Amino Acids (NEAA) and 1% sodium pyruvate (all from Gibco) was used for culture. Viral titer was determined by Plaque Assay^25^.

### Viral neutralization assay

Vero E6 cells were plated in a 96-well plate at 1.6 × 10^4^ cells/well in DMEM medium (Gibco) supplemented with 10% FCS, 1% P/S, 200 mmol/L L-glutamine, 1% MEM-NEAA, 1% sodium pyruvate (all from Gibco) and incubated overnight at 37 °C and 5% CO_2_. Five-fold serial dilutions of the constructs in medium were mixed with virus and incubated at 37 °C for one hour. Vero E6 cells were infected at a multiplicity of infection (MOI) of 0.06 with the construct/virus mixture at 37 °C. After 1 h, the construct/virus mix was removed, culture medium was added, and cells were incubated at 37 °C for 24 h. Mock cells represent uninfected Vero E6 cells, incubated with culture medium. After 24 h, cells were washed once with PBS and fixed with 4% paraformaldehyde (ChemCruz) at RT for 15 min. Following a washing step with PBS, fixed Vero E6 cells were permeabilized with 0.5% saponin (Roth) in PBS at RT for 15 min. Cells were blocked with a mixture of 0.1% saponin and 10% goat serum (Sigma) in PBS at 4 °C overnight. Vero E6 cells were incubated with a 1:1500 dilution of SARS-CoV-2 nucleocapsid antibody T62 antibody (Sino Biological, Cat.No. 40143-T62) in PBS supplemented with 1% FCS at RT for 2 h. Following washing steps with wash buffer (PBS supplemented with 0.05% Tween-20 (Roth)), the plates were incubated with a 1:4000 dilution of goat anti-rabbit IgG antibody, HRP conjugate (Merck KGaA, Cat. No. 12-348) in PBS supplemented with 1% FCS at RT for one hour. Following washing steps, TMB substrate (Invitrogen) was added to the wells and incubated at RT for 10 min in the dark. The reaction was stopped by adding 50 µl of 2 N H_2_SO_4_ (Roth) and colorimetric detection was performed at 450 nm and at 560 nm on an Infinite F200 multiplate reader (Tecan Group AG). The data was fit to a log(inhibitor) vs. response model with a variable slope in Prism v 9.2. (GraphPad).

### Pseudovirus neutralization assay

The commercial pseudovirus neutralization assay was performed at Tebubio. HEK293 cells stably expressing the full-length ACE2 receptor (Genbank #NM_021804.3) and pseudotype lentivirus produced with SARS-CoV-2 B.1.1.529 BA.1 variant Spike (Genbank #QHD43416.1 with B.1.1.529 BA.1 mutations) were used. These pseudovirions also contain the firefly luciferase gene driven by a CMV promoter, therefore, the spike-mediated cell entry can be conveniently measured via luciferase reporter activity. ACE2-HEK293 cells (BPS bioscience) are thawed, amplified in growth medium (BPS bioscience), then harvested and plated in white, clear flat bottom 96-well culture plates at 5 × 10^3^ cells/well in 50 µL of thaw medium. The cells are incubated overnight at 37 °C. The following day, visual control of cell layer homogeneity and integrity is validated using an inverted microscope. Test samples are diluted to reach 12× the expected final concentration in assay wells. The test samples are pre-incubated with Spike (SARS-CoV-2) pseudotyped lentivirus (BPS bioscience) or control bald pseudovirions (BPS bioscience) for approximately 30 min at room temperature. Finally, 10 µL of sample-pseudovirus mix are added in the assay wells containing the ACE2-HEK cells in 50 µL of medium. The IC50 was calculated in Prism v5 using a non-linear four-parameter regression fit.

### Transmission electron microscopy (TEM)

To visualize the hexameric ACE2-IgM-Fc, 5 µL of 0.02 mg/mL ACE2-IgM-Fc hexamer were pipetted onto a 200-mesh activated copper grid and incubated for 30 s. The samples were washed with 20 µL ddH2O and negatively stained with 5 µl of a 2% (w/v) uranyl acetate solution for 30 s. Excess solutions were removed with filter paper. TEM micrographs were recorded on a JEOL JEM-1400 Plus transmission electron microscope (JEOL) at 120 kV.

### Statistics and reproducibility

Individual measurements of the replicates are shown on the graphs. Mean values and standard deviations are calculated for *n* ≥ 3. The individual values for these calculations are provided in the Supplementary Data 1 file. The production and biophysical properties of the proteins were confirmed from biological replicates (transient cell transfection for protein production on different days and using different cell batches). The virus neutralization assays were performed in biological replicates and by using different batches of the proteins. The experiments are reproducible. The IC50s from ELISA and the virus neutralization experiments were calculated for each replicate in Prism v5 using a non-linear four-parameter regression fit.

### Reporting summary

Further information on research design is available in the Nature Portfolio Reporting Summary linked to this article.

## Supplementary information

Supplementary Information

Description of Additional Supplementary Files

Supplementary Data 1

Reporting Summary

## Data Availability

The numerical source data for the graphs and gel images are provided in the Supplementary Data 1 file. All other data are available from the corresponding author on reasonable request.
