# Supplementary material for: Multimeric ACE2-IgM fusions as broadly active antivirals that potently neutralize SARS-CoV-2 variants
Source: Commun Biol. 2022 Nov 12;5:1237. doi: 10.1038/s42003-022-04193-z (PMC9653429; doi:10.1038/s42003-022-04193-z)
Supplement: Supplementary file 1 — Supplementary Information [file 42003_2022_4193_MOESM1_ESM.pdf]

Supplementary Information

Multimeric ACE2-IgM fusions as broadly-active antivirals that potently neutralize SARS-CoV-2 variants

Hristo L. Svilenov[1,*], Romina Bester[2], Julia Sacherl[2], Ramona Absmeier[1], Carsten Peters[1], Ulrike Protzer[2,3], Carsten Brockmeyer[4], Johannes Buchner[1,*]

[1] Center for Protein Assemblies and Department Chemie, Technical University of Munich, 85748 Garching, Germany

[2] Institute of Virology, Technical University of Munich / Helmholtz Zentrum Munich, Munich, Germany

[3] German Center for Infection Research, Munich partner site, Munich, Germany

[4] Formycon AG, Martinsried/Planegg, Germany.

* To whom correspondence should be addressed:

hristo.svilenov@ugent.be

johannes.buchner@tum.de

phone: +498928913340; fax: +498928913345

Supplementary Figure 1

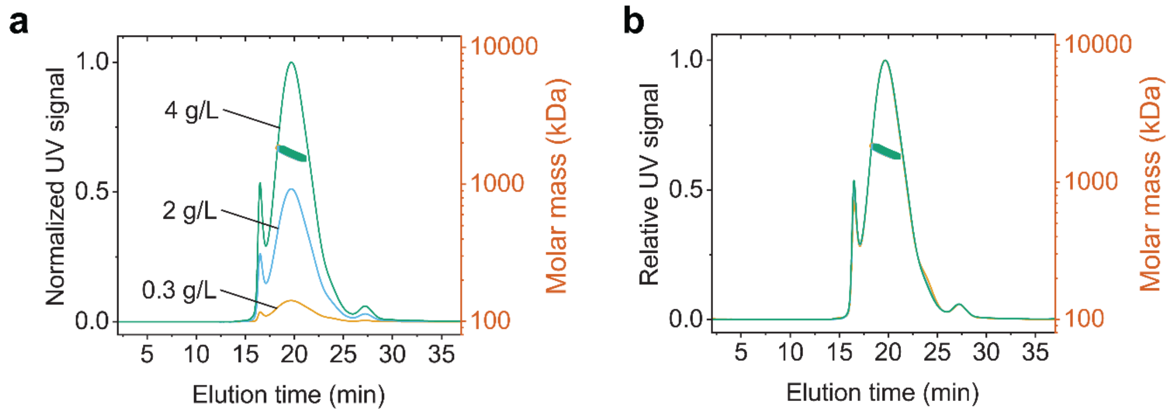

Supplementary Figure 1. ACE2-IgM-Fc hexamer composition at different concentrations analyzed by SEC-MALS. (**a**) Analysis of ACE2-IgM-Fc hexamer at different protein concentrations. (**b**) Overlay of the three chromatograms obtained at 0.3, 2 and 4 g/L ACE2-IgM-Fc hexamer. The samples for the analysis were concentrated with centrifugal filters with a 30-kDa MWCO and stored overnight at 2-8 °C before analysis.

Supplementary Figure 2

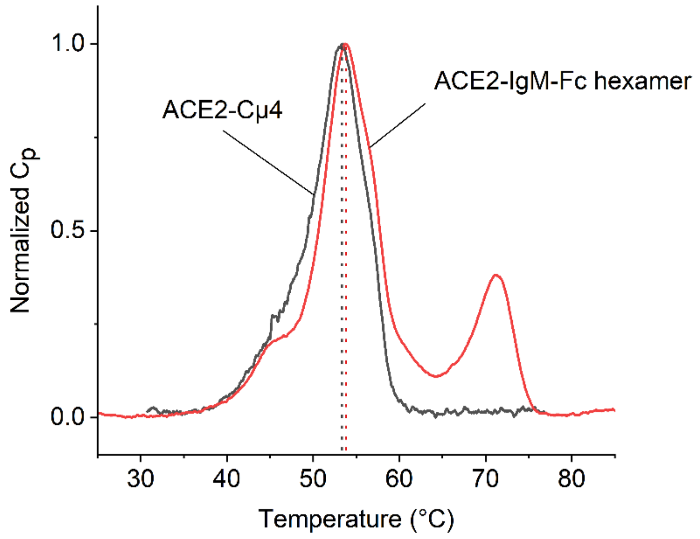

Supplementary Figure 2. Thermal stability analysis of the constructs by differential scanning calorimetry. Thermal unfolding profiles of ACE2-IgM-Fc hexamer (red) and ACE2-Cμ4 (black). The dotted lines indicate the melting temperatures for the ACE2 domains. The Cp is normalized to allow direct comparisons due to the difference in the molecular weight of the two constructs. The measurements were performed with a Microcal PEAQ-DSC system using 0.5 mg/mL protein in PBS. The temperature ramp was 1 °C/min.
